# Sociality and kinship constrain the free-mixing of pathogens in a wild mammal host population

**DOI:** 10.1098/rspb.2025.1242

**Published:** 2025-07-16

**Authors:** Clare H Benton, Richard Delahay, Barbara Shih, Rowland R. Kao, Robbie A. McDonald, Dave J Hodgson

**Affiliations:** ^1^ Animal and Plant Health Agency, Woodchester Park, Stonehouse, Gloucestershire GL10 3UJ, UK; ^2^ Department of Biomedical and Life Sciences, The University of Edinburgh The Roslin Institute, Edinburgh, UK; ^3^ Royal (Dick) School of Veterinary Studies, The University of Edinburgh, Edinburgh, UK; ^4^ Environment and Sustainability Institute, University of Exeter, Penryn, Cornwall, UK; ^5^ University of Exeter Centre for Ecology and Conservation, Penryn, Cornwall, UK

**Keywords:** badgers, bovine tuberculosis, whole-genome sequencing, kinship, *Mycobacterium bovis*

## Abstract

Pathogens rarely mix freely throughout host populations, and the presence of barriers to transmission can be detected as patterns of increased genetic isolation among pathogen isolates. Despite the importance of transmission patterns in host societies, and the risk of epizootics from wildlife disease systems, barriers to open pathogen transmission are poorly understood in wild hosts. We tested the influence of host kinship and social structure on genetic divergence among strains of *Mycobacterium bovis,* the causative agent of bovine tuberculosis (bTB), in a wild badger population. We measured genetic distances between *M. bovis* isolates from badger hosts that varied in their own genetic similarity (a proxy for kinship) and in their social group affiliations. Using jack-knifing analyses to control for pseudoreplication, we found that genetic distances between pathogen isolates decreased with increasing kinship of host dyads, but only when hosts shared the same social group. Our findings suggest that the open transmission of bTB in wild hosts is constrained by a combination of social and kin structure, in particular the sharing of similar pathogen strains among kin within social groups. We discuss the implications of these transmission structures for the understanding and management of wildlife diseases.

## 1. Introduction

Wildlife populations commonly harbour pathogens that cause infectious disease. Spillover of these diseases into other hosts can impact on human health [1,2], livestock industries [3,4], conservation [5,6] and companion animals [7,8]. The effective management of wildlife disease requires an understanding of how pathogen populations transmit through host populations, for example whether pathogens primarily transmit directly between individuals, indirectly via a shared environment or intermediate disease vector, or via a combination of processes. While the most basic transmission models for directly transmitted pathogens assume that host populations are free-mixing and every infected individual can infect every susceptible host at a constant rate [9], in reality all host populations are likely to be structured in ways that affect transmission pathways, the distribution of infected cases in space and time, and patterns of adaptive and stochastic genetic change in the pathogen population. Where barriers to transmission exist, evolutionary processes cause pathogens to diverge genetically, through either genetic drift or local adaptation. Using the molecular and statistical toolbox of landscape genetics we wish to determine the factors that slow or prevent the free-mixing of pathogens among hosts [10,11]. Such barriers or resistors are usually considered in terms of spatial distances and landscape features that limit the spread of infection by impeding the movement of infected hosts, or by preventing transmission events [11]. However, barriers to pathogen transmission may also result from the social behaviour of hosts [12] or through kin-biased infectious contacts [13,14].

In animal populations, the importance of social structure in transmission of infectious diseases is increasingly recognized [15–18], and the social organization of host populations can have a profound impact on the persistence of chronic diseases such as tuberculosis (TB) [19]. Prevalence of infection is expected to be higher for social species compared with solitary species [20], and for individuals in larger social groups [21], because social contact facilitates pathogen transmission [22,23]; however, the expected disease-imposed costs of group living can in some cases be mitigated by the presence of within-group social hierarchies [24]. When genetic distances among pathogen isolates are used to describe barriers to the free-mixing of directly transmitted pathogens, the expectation is that socially connected hosts will harbour more similar pathogen genotypes [25,26], while social disconnection will be associated with greater genetic distances between pathogens [27]. Genetic distance can increase with social isolation of hosts either through exposure of isolated host groups to different pathogen strains at the point of infection, through microevolutionary divergence between isolated strains, or their combined effects.

As well as being spatially and socially structured, host populations are frequently genetically structured by patterns of relatedness among individuals and the interplay between pathogen spread across landscapes, and the genetic structure underpinning host populations can have important implications for the management of disease [28]. For example, social groups of individuals may be highly related if juveniles do not disperse (natal philopatry) [29], while dispersal events, both permanent (individuals permanently leaving their natal group) and transient (individuals mating outside of their social group via temporary excursions), can weaken the association between social and kinship structures. Within social groups, age, sex and kin-biased associations can add further heterogeneity to contact rates among individuals [18] and may affect disease dynamics at wider scales [30]. Kin-biased transmission of pathogens has been suggested within socially organized wild animal populations by virtue of increased infection risks posed to individuals with infected relatives [14]; for example in social groups of the European badger (*Meles meles*) badger cubs related to infectious adults within the social group experienced a higher risk of infection as compared with cubs that were unrelated to infectious adults in the group [13]. In the rare examples where both host and pathogen genotypes are available and relatedness is linked to spatial/social proximity, kin are expected to harbour more similar pathogen genotypes [31], due either to kin-biased social transmission (i.e. relatives spending more time together), or to genetic associations between pathogen infectivity and host susceptibility (i.e. a heritable component of susceptibility to infection) [32,33]. Genetic relationships between individual hosts are best assigned by the construction of pedigrees, but a common proxy is to measure genetic distance among host individuals to estimate relatedness [34,35].

Bovine tuberculosis (bTB; caused by infection with *Mycobacterium bovis*) has far-reaching economic, animal welfare and human health impacts in many parts of the world. As the pathogen is capable of infecting a wide range of mammal species, there are many settings globally where infection of wildlife populations creates challenges for disease control in cattle [36]. In the UK, badgers (*Meles meles*) have long been considered as the main wildlife reservoir of *M. bovis* [37,38]. The potential for badger populations to maintain infection for protracted periods was confirmed by the first study using whole-genome sequencing to directly estimate within- and between-species transmission, which demonstrated high levels of *M. bovis* transmission among badgers and the long-term maintenance of a single lineage [39].

Our understanding of barriers to the transmission of bTB in badger populations has been informed by comparison of pathogen isolates in hosts within and among social groups. If the organization of many badger populations into discrete territorial social groups limits disease spread [40–42], then we would expect *M. bovis* strains in hosts belonging to the same group to be more closely related than those in hosts from different social groups. This is borne out by random forest models confirming that social group membership is a strong predictor of *M. bovis* genetic distance in badgers [39]. Within badger social groups, the presence of infectious adult female badgers was found to be associated with new infections [40], particularly in cubs [43], and the risks of infection in badger cubs increased with the number of infected group members rather than total group size [13]. Analyses of badger social networks have found links between network position and TB status highlighting the importance of social structure to pathogen epidemiology [44] with some individuals disproportionately contributing to pathogen flow between groups due to their network position. In an undisturbed badger population proximity loggers revealed that interactions within social groups accounted for more than 90% of all contacts, highlighting the marked impact of social group membership on contact rates [45].

The evidence base described above suggests that social-group membership is the main structuring force for bTB transmission via direct contact between hosts, but other potential barriers to open transmission exist, including sexual and demographic structure of the population, and kinship. There is some indirect evidence that kin structure within social groups influences transmission risks, as cubs are more likely to be infected if related group members are excreting the pathogen [13]. However the relative importance of social structure versus kin structure on *M. bovis* transmission in badger populations is not known, and coupling badger genotype data with pathogen sequence data offers a rare opportunity to investigate transmission barriers caused by kinship and demography.

In high-density populations, such as are found in parts of the UK, badgers live in social groups made up of three to eight individuals on average [46]. Many badgers remain in their natal group throughout their lives [47,48], resulting in marked kin structure within social groups [49], but even in such highly structured populations some badgers will move between groups [47] and high levels of extra-group mating dilute patterns of within-group relatedness [50–52]. Studies of dispersal within badger populations differ in their estimates of the frequency and magnitude of dispersal events [53–55] and in their findings related to sex-biased dispersal [56,57]. Badger social groups will therefore comprise a mixture of related and unrelated individuals with the group composition (and therefore relatedness structure) changing over time and in response to local conditions (such as fluctuations in population density, both natural and as a result of population management [58,59]. This imperfect association between social and kin structure provides the opportunity to test whether kinship adds an additional barrier to the free-mixing of pathogen genotypes.

To determine the main barriers to free-mixing of pathogen genotypes in badger populations and characterize their respective contribution, we examine genetic distances between *M. bovis* isolates obtained from genetically similar and dissimilar individual badgers residing in the same or in different social groups, alongside measures of age- and sex-differences between hosts. There are few examples of study systems of wildlife where coupled host and pathogen genetic data allow for such an examination of factors predicting pathogen spread; one notable exception is a study of the transmission of feline immunodeficiency virus in a puma population where both host spatial proximity and host relatedness predicted pathogen spread [31]. Based on previous findings from the same badger population where kin structure predicted infection risk within social groups [13] and where social structure predicted genetic distances between pathogen isolates [39], we predict shorter genetic distances between *M. bovis* isolates collected from pairs of badgers within groups, compared with non-social pairs. Furthermore, we predict that the relatedness of the badger hosts will also have influence, with pathogen genetic distances decreasing as host relatedness increases. We also predict that pathogen genotypes will diverge with increasing time between sampling due to genetic drift, but we do not have clear predictions regarding transmission barriers caused by sex- and age-related differences between host individuals.

## 2. Methods

### (a) Study area

**Figure 1.**
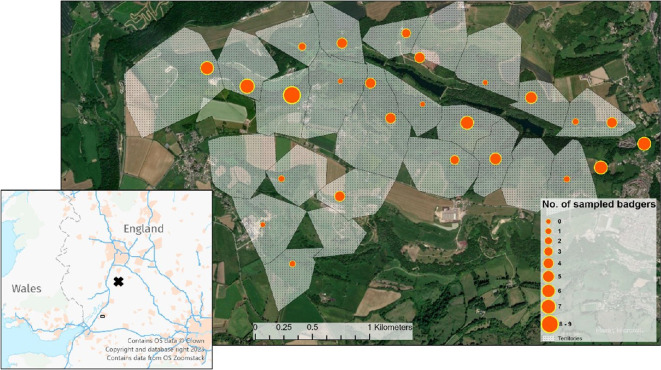
Locations of the main setts (burrow systems) within badger social group territories where individuals contributing to the analyses in this article were captured, scaled by the number of *M. bovis* isolates obtained. Social group territorial boundaries as delineated by bait-marking activities in 2004 are shown for illustrative purposes, noting that there are annual variations in territory boundaries. Inset shows the wider geographical context of the study area. Territory mapping by bait-marking only happens around setts that are active in a given year whereas badgers that were included in this study have capture histories spanning 1993–2019. For this reason, some territories shown do not include badgers from the present study and some badgers are shown at sett locations that were not bait-marked in 2004 as they were not active at that time.

The study area is located at Woodchester Park on the Cotswold escarpment in Gloucestershire, southwest England (2°16′E, 51°43′N,) and comprises a wooded valley surrounded by pastoral and arable farmland [60]. The resident badger population has been the subject of a long-running capture-mark-recapture project, providing insights into the epidemiology of *M. bovis* infection in badgers in this region of endemic infection in cattle [61]. On first capture, badgers are permanently marked by means of an abdominal tattoo to permit long-term identification. The majority of badgers in the study system are first caught as cubs (i.e. in their first year of life) therefore it is possible to accurately determine their age in years throughout their capture history. This is not possible for the far smaller number of badgers that are first caught as adults (of the 108 individual badgers that contributed to the analyses below, 97 (90%) were first caught as cubs and therefore were of known age). Badgers are trapped at active setts (burrow systems) throughout the study area (figure 1), and each year a bait-marking exercise (see [40]) is carried out to allocate setts to social group territories. Captured badgers can then be allocated to a social group at each capture event based on where they were caught. The number of social groups is not static and variations in social group number over the study period ranged from a minimum of 17 to a maximum of 24 during the study period. Active badger setts in the study area have been routinely trapped and animals sampled for *M. bovis* up to four times annually (full details described in [62]). For the purposes of the present study, we consider only those infected individual badgers that had been genotyped and from which *M. bovis* was cultured and whole-genome sequenced. Culture-based diagnostics have low sensitivity [63], hence applying these filters to the entire database of Woodchester capture records restricted our analyses to 108 culture-positive individuals from which *M. bovis* isolates were collected between 2010 and 2019. As culture-positive badgers were frequently caught more than once over their life history, 53 of the 108 badgers contributed more than one *M. bovis* isolate to the analyses.

### (b) Host genotyping and relatedness estimation

On the first capture of each individual badger, a hair sample was routinely taken (approx. 20−30 hairs plucked from the rump) and stored in 80% ethanol before being submitted for DNA extraction and genotyping [50]. Full details on genotyping are available elsewhere [52]; however, in brief, individuals trapped between 1993 and 2002 were genotyped based on the DNA extraction protocols set out in Carpenter *et al*. [50] while hair samples from individuals trapped after this period were genotyped at the NERC Biomolecular Analysis Facility (University of Sheffield), as described in Marjamäki *et al*. [52]. As hair genotyping had taken place in batches, cross-validation protocols were used, including re-genotyping a subset of subsamples; this is described in detail elsewhere [52]. Genotype data from 108 individual badgers were used in the study (51 males, 57 females from 25 different social groups, trapped between 1993 and 2019 inclusive). Details of capture histories of the badgers included in this study are shown in electronic supplementary material, figure S1.

We used 20 microsatellite markers to derive genotypes, each with four to seven alleles [64]. Additional information on marker performance and comparison of relatedness estimators is available in the electronic supplementary material. Pairwise relatedness between badgers was therefore estimated between all pairs of individuals in the study (*n* = 108) using the Queller and Goodnight rxy estimator [65]. The expected rxy relatedness coefficients between individuals based on relationship are as follows: parent/offspring and full sibling 0.5, half-sibling 0.25 and unrelated 0 [66]. Negative values of rxy may occur if gene frequencies of the two compared individuals differ from the population mean in opposite directions [65]. The relatedness coefficients allowed the assignment of categorical relationships between pairs of badgers (identifying full and half-sibling, etc.); however, simulation testing suggested that the power of 20 microsatellite markers to accurately differentiate between relatedness classes was likely to be limited, hence relatedness coefficient was used as a continuous variable in the analyses.

### (c) Pathogen genotyping

Clinical samples routinely collected from anaesthetized badgers during sampling included oesophageal and tracheal aspirates, urine, faeces and swabs of bite-wounds or abscesses. Electronic supplementary material, figure S1 shows the culture sampling histories of the 108 badgers included in this study. These samples were processed and seeded onto media selective for *M. bovis*. When bacterial growth was observed (this may take 6−12 weeks), a single colony was inoculated in HPLC water and spoligotyped; the remaining growth was harvested into two tubes, each containing 1 ml of 7H9 Middlebrook broth medium with 20% glycerol, and stored at −20°C. A total of 241 isolates from 108 badgers collected between 2000 and 2019 inclusive were recovered from the archive. Slopes of modified 7H11 media [67] were seeded with the defrosted samples in October 2020 and incubated at 37°C for a six-week period for regrowth under containment level 3 conditions. Successfully regrown isolates were heat killed in hot blocks at 80°C for 30 min and standard Illumina protocols (NexteraXT kits) for generating libraries from heat-killed cell suspensions were applied. These were then run on the Illumina NextSeq instrument with 2 × 150 bp paired-end reads. Further detail on bioinformatics protocols and single nucleotide polymorphism (SNP) calling are included as electronic supplementary material.

Pairwise genetic distances (defined as the raw number of sites that differed in their genetic profile at 1569 quality filtered SNPs) between *M. bovis* isolates were calculated in R (v. 4.0.2) using the package ‘ape’ (v. 5.6−2) [68].

### (d) Data analysis

Analyses were carried out using R software version 4.5.0 [69]. The impact of social group membership and host genetic population structure on the genetic distance between *M. bovis* isolates was analysed by constructing mixed models using the R package ‘lme4’ [70]. This was coupled with jack-knifing procedures to remove biases and pseudoreplication associated with the multiple representations of pathogen isolates and host individuals in the measurement of pairwise distances [71]. In reviews of statistical methods to deal with the inherent pseudoreplication found in dissimilarity matrices, jack-knifing by ‘population’ (here, host and pathogen isolate identity) has been found to be superior to other methods, especially following critique of the classic Mantel-testing approach [72–75]. Genetic distances among badger genotypes were based on differences in alleles found across a set of microsatellite markers and estimated using the Queller and Goodnight rxy estimator of relatedness [65] in the R package ‘related’ [76].

Each badger’s main social group was defined as the group that it had been resident in for the longest period of its capture history (according to trapping records) as this was found previously to be the best spatial predictor of genetic distance between isolates by Crispell *et al.* using a smaller dataset from the same population [39]. In the majority of cases, this was also the individual’s natal group (of the 97 badgers first caught as cubs, 92 (95%) were recaptured in the same social group as they were assigned to at their first capture, i.e. their natal group). Main social group is therefore, in the vast majority of cases but not exclusively, analogous to natal social group.

Genetic distances for pairwise combinations of *M. bovis* isolates were categorized according to whether the badgers from which they were collected shared the same or different social groups in the year when the isolate was collected. Pairwise distances were also categorized according to the age classes of the badgers as follows: comparisons between isolates from two adult badgers (‘Adult-Adult’), between an adult and a cub or yearling (‘Adult-Young’), and between pairs of cubs/yearlings (‘Young-Young’). Data were restricted to only include pairwise comparisons where isolates had been collected within 3 years of one another, in order to only include individuals likely to have been in the population at the same time. This is in line with recent estimates of median survival time of badgers in this population as 3.6 years [77]. We also categorized pairwise isolate distances according to the sex of the host badgers. To account for temporal divergence in pathogen genotypes (consistent with the molecular clock hypothesis [78]), the number of years between isolate collection dates was also included in the model as a fixed effect.

To test for an effect of social group membership and kin structure on *M. bovis* isolate distances, we constructed a mixed effects model using the R package ‘lme4’ [70] with genetic distance between pathogen isolates as the response variable. Genetic dissimilarity between hosts (modelled as a continuous variable using the rxy relatedness estimator [65]), age-class comparison, sex-based comparison (M : M, F : F, M : F), time between isolate collection dates and social group membership (whether the badgers were in the same social group at the time of sampling) were all included as fixed effects. A term was also included for the interaction between host genetic similarity and social group membership. As individual badgers contributed multiple dyadic data points to the response variable, the identities of the badgers in each pairwise comparison were included as random effects in the model. Counts of SNP differences in the response variable were treated as Poisson-distributed.

A jack-knifing procedure was used to remove bias from the mixed model’s estimates of fixed effects caused by pseudoreplication due to multiple representations of individual hosts and pathogen isolates in pairwise comparisons [71]. Jack-knifing removed each pathogen isolate in turn from the model and refitted to calculate pseudo-values and then jack-knifed estimates of the variance components and fixed effects of the model. These estimates provided jack-knifed 95% confidence intervals for the parameters of interest. Two isolates collected from badgers in the study area exhibited unusually large genetic distances as compared with the remainder of the isolates (electronic supplementary material, figure S2). These isolates are known to be derived from cattle herds (as evidenced by their closest ancestral isolate on the wider phylogenetic tree being from a cow in the study area rather than a badger; also by their very high genetic distance from other isolates as determined by SNP differences): these were removed from the dataset prior to analysis.

## 3. Results

Both a host and pathogen genotype were available from 108 individual badgers (see electronic supplementary material, figure S1). Where multiple genetically distinct *M. bovis* genotypes were available from an individual badger (*n* = 53 badgers with more than one genotype available) these were included in analyses of genetic distance between, but not within, badger hosts as this was outside the scope of the present study. The remaining pairwise comparisons of *M. bovis* isolates yielded 8530 measures of genetic distance; of these, 376 were between badgers from the same social group and 8154 were between badgers from different groups (electronic supplementary material, figure S2).

**Figure 2.**
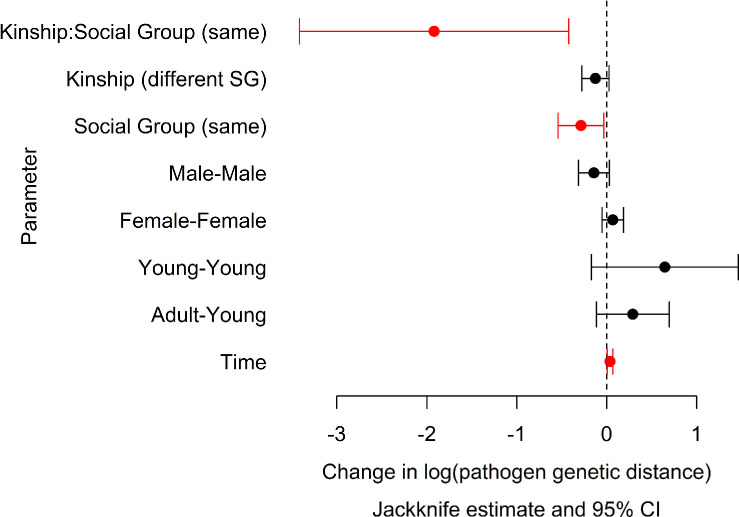
Jack-knifed estimates and 95% confidence intervals for the effect of predictors on genetic distances between *M. bovis* isolates. All estimates are differences from the reference category (adult male–adult female dyads of average kinship residing in different social groups), caused by being from the same social group, increasing host genetic similarity, separation in time (in years), being in different age groups or sex groups, and the additional effect of kinship in badger dyads sharing the same social group. Those 95% confidence intervals that do not span zero are shown in grey and represent increasing genetic distance with increasing separation in time, social group membership (badgers in same social group predicted to have shorter genetic distances between isolates) and host relatedness (isolate distance decreasing as relatedness between hosts increases.

Pairwise genetic distances between *M. bovis* isolates increased significantly with the interval between isolate collection years, at a rate of approximately one additional SNP difference per year. Comparisons of *M. bovis* isolates in dyads that included young badgers (cubs and yearlings) showed generally greater genetic distances than those between adults; however, none of these age-group differences were significant (confidence intervals spanning zero in figure 2; table 1). While male dyads tended to have shorter genetic distances between isolates than female dyads, this difference was not significant (confidence intervals spanning zero in figure 2; table 1).

**Table 1.** Summary of estimates from a jack-knifed model to test for an effect of social group membership and kin structure on *M. bovis* isolate distances while accounting for sex and age structuring. The reference category is the expected log(number of SNP differences) between two *M. bovis* isolates sampled in the same year from an adult male and an adult female, of average kinship and residing in different social groups. All other estimates are deviations from this reference, caused by membership of different categories or by a unit increase in kinship measured by the rxy relatedness estimator [65]. Estimates with 95% confidence intervals that do not span zero (emboldened) are considered significant.

model term	jack-knifed estimate	95% confidence intervals
intercept	2.34	2.05, 2.62
**social group (same**)	**−0.29**	**−0.54, −0.03**
host relatedness	−0.13	−0.28, 0.03
**time (year**)	**0.04**	**0.01, 0.07**
adult/young	0.29	−0.11, 0.70
young/young	0.65	−0.17, 1.46
F/F	0.07	−0.05, 0.19
M/M	−0.06	−0.32, 0.03
**social group (same) and increasing relatedness**	**−1.92**	**−3.42, −0.43**

**Figure 3.**
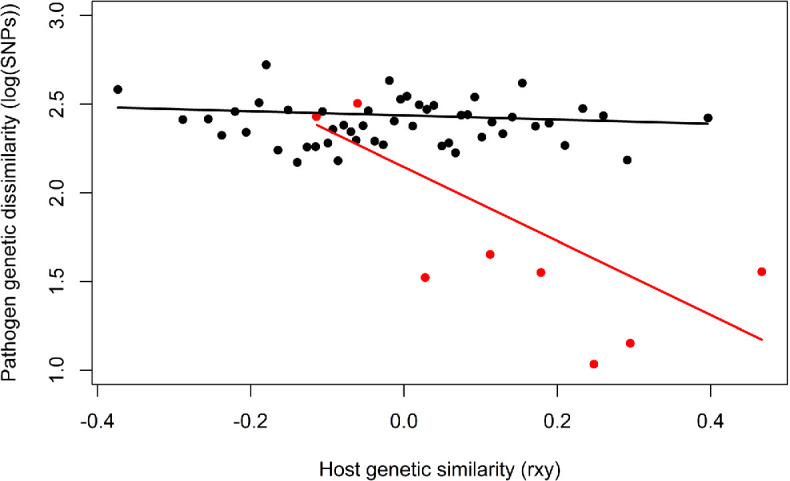
Relationship between pathogen pairwise genetic dissimilarity and host pairwise genetic similarity, for *M. bovis* isolates collected from Woodchester Park badgers that occupy the same (grey) or different (black) social groups. Fitted lines use jack-knifed parameter estimates, assuming no time difference between isolates. Data are derived from electronic supplementary material, figure S2*,* with host kinship binned into equal-sized samples along the x-axis (and plotted at the midpoint of each bin), and pathogen genetic dissimilarity measured as the log of the number of SNP differences for each pairwise comparison, then averaged in each bin.

The jack-knifed mixed-effects model revealed significant differences in the genetic distance between *M. bovis* isolates from pairs of badgers relative to the interaction of social group membership with host genetic similarity, and the temporal separation of the isolates. (figure 2, table 1). The pathogen isolate dyads that were genetically closest were derived from genetically similar badgers sharing the same social group (figure 3). With time interval set to zero, pathogen isolate dyads collected from badgers in different social group and from less related badgers in the same social group were predicted to be separated by around 12 SNPs as compared with isolates collected from the most related badgers within the same social group that were predicted to be as little as three SNPs apart (figure 3).

## 4. Discussion

We used pairwise genetic distances between *M. bovis* isolates to determine whether kinship, social structure, sex or age, act as barriers to the free-mixing of the pathogen through a wild population of badgers. As expected from ecological and microevolutionary processes of pathogen turnover and drift, pathogen genotypes diverged as the time interval between isolates increased, with a molecular clock rate of approximately one SNP per year, consistent with previously published estimates for *M. bovis* [79,80]. As predicted, the genetic similarity of pathogen isolates was influenced by both kinship and shared membership of social groups. The social group effect is consistent with the contention that the stable social structure of undisturbed badger populations may act as barrier to *M. bovis* transmission (see [40]). Notably the effect of kinship was only observed in host dyads that shared the same social group, indicating that kin structures present additional barriers to pathogen flow, limiting transmission between less related badgers within social groups. These results infer that transmission of infection occurs mainly among family members that share social group territories. In contrast, pathogen genetic isolation is just as great between non-kin hosts within social groups as it is between badgers (of any kinship status) from different groups, suggesting that inter-family or inter-territorial interactions are not the main routes of transmission of infection. We find no evidence for age or sex-related barriers to pathogen transmission.

Although our models of pathogen genetic isolation have identified some drivers of transmission, broad heterogeneity remains in genetic isolation among host-and-pathogen dyads. The fact that pathogen isolates can be closely related between unrelated hosts residing in different social groups (see electronic supplementary material, figure S2) concurs with evidence that extra-territorial excursions are not uncommon and probably relate to the search for mating opportunities [52]: pedigree-based estimates have suggested that 37%–50% of the cubs in this study population may be sired by a male from outside their natal group [50,52].

Our findings contrast with those from a recent study carried out at a larger landscape scale in Northern Ireland, where estimated relatedness between badgers (based on microsatellite markers) did not predict *M. bovis* isolate genetic distances [28]. This may relate to the considerable differences between the studies including their spatial scale (100 km^2^ in the Northern Ireland (NI) study compared with approximately 7 km^2^ in the present study); host population density (approximately 4 badgers km^−2^ in NI compared with 24 badgers km^−2^ in Woodchester Park between 2000 and 2019) and the management context (the population in NI was undergoing selective culling whereas the Woodchester Park population was largely unimpacted by management interventions for the majority for the study period). While the lack of relationship between badger population genetics and the partitioning of pathogen genetic distance in NI was used to suggest that badger-to-badger transmission did not play a major role in transmission dynamics, the authors acknowledged that factors such as population density and population management may have obscured any associations [28]. Our results show that while social structure appears to be the strongest barrier to pathogen transmission in our study, kin structure within social groups in a high-density, largely undisturbed badger population acts as an additional barrier to pathogen transmission. Two rival mechanisms might explain the genetic similarity of pathogen isolates among host kin within social groups. First, preferential social interaction with kin (e.g. via allogrooming; kin-biased biting and fighting; parental care) can result in the restriction of pathogen circulation to family groups, while genetic drift or localized forces of selection cause genetic divergence of more physically isolated strains. Contact rates are likely to be higher, particularly between cubs and their mothers and between members of the same litter. Behaviours that bring badgers into very close contact, such as play-fighting and mutual grooming, have been linked to enhanced risk of acquiring TB in meerkats [81] and might occur preferentially between relatives. In badgers the principal site of *M. bovis* infection is thought to be the lungs following inhalation of infectious aerosol particles [82], therefore behaviours which bring individuals into very close proximity are likely to incur a higher transmission risk. However, there is very limited available information on how these behaviours correlate with kinship; the only example finding that allogrooming in badgers was *not* associated with pairwise relatedness [83]. Studies using proximity collars have suggested that related adult badgers spend more time together than with unrelated animals; however, as collars cannot be fitted to badger cubs for welfare reasons, contact patterns between cubs and adults based on relatedness remain unknown [84]. Although a wealth of observational work has been carried out on badger behaviour [48,85–87] including valuable insights from using proximity collars to capture interactions between individuals [45], few studies have taken account of kin structure [88], hence at this stage we cannot verify the mechanisms that cause the observed barriers to pathogen transmission.

An alternative potential driver of kinship effects on pathogen genetic distances might relate to genetic associations between pathogen infectivity and host susceptibility. In other words, pathogen strains might be locally adapted to host genotypes, resulting in barriers to transmission to non-kin. While a small heritable component of susceptibility to infection with *M. bovis* has been found in badgers, environmental factors (such as social group environment in early life) are thought to be more influential than genetics [89]. It may be the case that particular relationship types are of greater importance in *M. bovis* transmission within badger social groups (e.g. mother–offspring) (see [40]). However, our ability to accurately differentiate these relationships either by pedigree construction [89] or microsatellite-based estimators is limited by the number of microsatellites available. An extension of this work would be to explore the genetic population structure of the host in greater depth using SNP-based methods which may further elucidate routes of transmission that are of particular importance.

Our findings have implications for the management and surveillance of bovine TB in badger populations. In the UK and Ireland badgers have been culled as part of measures to reduce the risk of *M. bovis* transmission to cattle. The disruptive effects of badger culling on social structure have been widely documented and linked to opportunities for the wider spread of infection [90]. In the present study, we have begun to quantify the impact of badger social structure on expected pathogen distances which has useful applications to the targeting of ongoing surveillance of *M. bovis* in badger populations. For example, if surveillance of *M. bovis* genetic diversity in badger populations was necessary and samples were collected from badgers within the same social group this may lead to an underestimation of the actual diversity within the wider population. In order to better capture the pathogen genetic diversity present in a badger population it would be necessary to sample from different social groups rather than multiple individuals within the same groups. Badger culling also impacts on badger genetic population structure and relatedness [59]; our study shows that this may also affect transmission patterns and pathogen genetic diversity. We would predict that as culling impacts badger social structure by increasing inter-group movement [91] and reducing the integrity of social group territories [60] that the disparity we have observed in pathogen distances between members of the same social group as compared with members of different social groups would weaken and potentially eventually be undetectable. Furthermore, we would predict that as kin structures break down, the observed relationship between host relatedness and pathogen relatedness would also weaken and eventually become undetectable. Badger vaccination is another management intervention being used to reduce the risk of cattle acquiring bovine TB from badger populations [92,93]. Field studies indicate that vaccination of a sufficient proportion of badgers in a social group confers indirect protective effects to unvaccinated group members [94]. Our observations of the importance of group membership and kinship within groups in driving transmission highlight the potential benefits of this phenomenon for disease control.

Our findings should motivate broader consideration of the influences of both kinship and social structure in the population genetics of pathogens of wildlife. Host species will vary in the relative importance of spatial, social and kin structure to the transmission of infectious disease. Transmission pathways will often be difficult or impossible to observe in the wild, hence the analysis of host and pathogen population genetics offers a powerful forensic tool. Barriers to the free-mixing of pathogens in host populations can fundamentally change the dynamics of infection, with major implications for pathogen persistence, risks of spillover into other species, and the likely effectiveness of disease control interventions.

## Data Availability

Data and code for the analyses in this paper are available at the Dryad Digital Repository [95]. Supplementary material is available online [96].
